# Determining the role of music attitude and its precursors in stimulating the psychological wellbeing of immigrants during COVID quarantine – a moderated mediation approach

**DOI:** 10.3389/fpsyg.2023.1121180

**Published:** 2023-07-14

**Authors:** Xiaokang Wang, Wenrong Huang

**Affiliations:** College of Music and Dance, Guizhou Minzu University, Guiyang, Guizhou, China

**Keywords:** social cognitive theory (SCT), music and cognition, music attitude precursors, psychological well-being, immigrants, music in COVID quarantine, moderated-mediation

## Abstract

Based on social cognitive theory (SCT), the purpose of this study is to examine the role of music attitude and its essential precursors in stimulating the psychological wellbeing of immigrants in isolation (quarantine) during the COVID pandemic. This study employed quantitative methodology; an online survey was administered to collect sufficient data from 300 immigrants who traveled to China during the pandemic. Data were collected from five centralized quarantine centers situated in different cities in China. Additionally, the valid data set was analyzed using structural equation modeling (SEM) via AMOS 24 and SPSS 24. The results indicate that potential predictors such as cognitive - music experience (MEX), environmental - social media peer influence (SPI), and cultural factors such as native music (NM) have a direct, significant, and positive effect on music attitude (MA), which further influences immigrants’ psychological wellbeing (PW) during their quarantine period. Moreover, in the presence of the mediator (MA), the mediating relationships between MEX and PW, and NM and PW, are positive, significant, and regarded as partial mediation. However, the moderated mediation effects of music type (MT) on MEX-MA-PW and NM-MA-PW were found to be statistically not significant and unsupported. This study contributes to the literature on the effectiveness of individuals’ music attitude and its associated outcomes, focusing on mental health care in lonely situations such as quarantine during the COVID pandemic. More importantly, this study has raised awareness about music, music attitude, and their beneficial outcomes, such as mental calmness and peacefulness for the general public, particularly during social distancing, isolation, and quarantine in the COVID pandemic situation.

## 1. Introduction

In response to the emergence of COVID-19, declared a global pandemic by World Health Organization (WHO) on February 11th, 2020, countries imposed strict lockdowns to curb its spread, closing all schools, businesses, and offices (Tabish, 2020; Wu et al., 2020). This forced global citizens to stay at home, which was effective in preventing the spread of the virus but may have had negative psychological impacts such as depression and anxiety among them (Riaz et al., 2021). Prior research has identified that psychological problems such as distress, frustration and anxiety are the normal responses to an unpredictable and threatening situation, e.g., the COVID pandemic (Putter et al., 2021; Loveday et al., 2022). As it has been observed that during COVID-19 the psychological effects of the pandemic were exacerbated by the necessity to remain in social isolation to prevent the spread of COVID-19 (Carlson et al., 2021). But people throughout the world adopted various strategies to cope with the negative psychological effects of the COVID pandemic. People’s engagement with music and music experiences was one of the most effective ways they used to improve their psychological well-being (Loveday et al., 2022). Even at the early stages of the pandemic, music played a significant role in curing stress and anxiety (Putter et al., 2021). Studies have shown that daily exposure to music can provide comfort and solace during uncertainty and stress period (Biasutti et al., 2021; Fink et al., 2021). As it has the ability to evoke emotions and create a sense of community. But how can music impact the well-being of an individual, particularly among immigrants during their isolation and quarantine time period need to be investigated.

Various studies suggest that listening to music relaxes the body and provides a sense of social comfort (Tan and Miksza, 2019; Hennessy et al., 2021). Moreover, people can choose music as the best tool to safeguard themselves from situations that create anxiety in their daily activities by engaging with its lyrics and beats. Research has found that music is more effective than other leisure activities in achieving an individual’s well-being (Hennessy et al., 2021). Therefore, music is known as the “soul of life” and the “peace of mind” due to its strong emotional power (Cardona et al., 2020). Listening to music is one of the most pleasurable activities in human life and is mainly done to generate and regulate emotional experiences (Västfjäll et al., 2012; Loveday et al., 2022). This function of music to regulate emotions is not restricted to any particular culture or nationality; it has been evident across nationalities and multiple cultures that music is used to self-regulate emotions when individuals undergo any emotional experience (Tan and Miksza, 2019). Yo-Yo Ma, a famous musician, stated that “What the pandemic has crystallized in my mind is that we need music because it helps us get to very specific states of mind” (Ferreri et al., 2021).

Studies have begun to investigate the effects of music (Musgrave, 2022), music teaching (Tang and Corrado, 2022), music engagement (Rosenberg et al., 2021), musicians’ performance during COVID-19 (López-Íñiguez et al., 2022), popular music lyrics during COVID-19 (Putter et al., 2021), anxiety and depression among musicians (Loveday et al., 2022), etc. However, the present study aims to explore how music attitude and its precursors affect the psychological well-being of immigrants during the COVID quarantine period, providing a new perspective in the literature on music. While other studies are limited to specific contexts and situations (Du and Leung, 2022; López-Íñiguez et al., 2022), this study reflects the broad experiences of immigrants during their quarantine period. The study will highlight the impact of music attitude factors that may further affect psychological well-being during COVID quarantine. One of the recent studies found music type to be an independent variable (Flynn et al., 2022), and music and its types have also been utilized to investigate individuals’ preferential behavior (Kim and Kang, 2021; Greenberg et al., 2022). However, the current study emphasizes examining the moderating effect of music type on individual psychological well-being.

In addition, there is very limited research on the relationship among MEX, MA, and PW (Musgrave, 2022). Music experience refers to the way individuals perceive, feel, and interact with musical sounds (Van der Hoeven and Hitters, 2019). It encompasses the way music affects one’s mood, emotions, sensations, and cognitive and cultural interpretation of sounds and melodies. Music experience is unique to each person and can be influenced by factors such as personal taste, cultural background, and the context in which the music is heard (De Witte et al., 2020). A study on music education has been conducted by Du and Leung (2022), but the current study contributes to a new direction in understanding how MEX enhances listening, downloading, and storing music, which may further influence the mental healthcare or wellbeing of immigrants during the COVID quarantine period. Moreover, this study includes a robust variable named “native music,” which refers to the traditional, cultural music of a particular group of people or place, often associated with their history and heritage (Campbell, 2021). Native music is typically passed down orally from generation to generation and may feature instruments and musical styles unique to the group. It is an essential part of the cultural identity of a people and can serve as a source of pride and connection to their roots (Mbugua, 2022). As native music feels calm and relaxing because of its lyrics, tune, and rhythm for individual. Therefore, Cowen et al. (2020) state, “Music is a universal language, but we do not always pay enough attention to what it’s saying and how it’s being understood.” Similarly, social media peer influence (SPI) is a substantial variable that can play a significant role in achieving psychological well-being during COVID quarantine. SPI refers to the impact that one’s peers (friends, family, and other social network members) have on an individual’s thoughts, feelings, and behaviors through the use of social media platforms. It describes the ways in which people are influenced by the opinions, attitudes, and behaviors of others connected via social media (Riaz et al., 2021).

Apart from content analyses of specific music attitudes and wellbeing during COVID quarantine, recent studies have demonstrated that music has the ability to significantly reduce psychological suffering (De Witte et al., 2020; Ferreri et al., 2021; Loveday et al., 2022). Engaging with music can calm the nervous system and help to alleviate stress (López-Íñiguez et al., 2022). According to music psychologists, listening to music can help people relax, feel good, and express or regulate their emotions (Kim and Kang, 2021). Another study found that people began listening to more happy and unfamiliar songs during the pandemic because they believed that listening to happy music could change their sad mood to a happier one (Granot et al., 2021).

The present study provides new insights into the relationship between music, music attitude, and their positive outcomes, such as mental peace and calmness for the general public, particularly during social distancing, isolation, and quarantine periods. While previous literature has explored the relationships between music and wellbeing (Loveday et al., 2022), the current study contributes a novel direction by examining the relationship between crucial factors, such as music experience, social media peer influence, and native music, with music attitude. This more comprehensive understanding can have a more significant impact on improving mental health, especially for immigrants with diverse language, culture, and nationalities, during the COVID quarantine period. Moreover, this research aims to raise awareness about music attitude and its potential benefits in the form of mental calmness and peacefulness among the general public, particularly during social distancing, isolation, and the COVID pandemic situation. Based on the foregoing discussions, this study aims to explore the answers to the following important research questions:

1. How does music attitude and its precursors influence immigrants’ psychological well-being during the COVID quarantine period?
2. Whether immigrants’ music attitude mediates the relationship between the factors (music experience and native music) and their psychological wellbeing during quarantine in COVID pandemic situation?
3. Whether music type moderates the mediating relationship between the factors (music experience and native music) and immigrants’ psychological wellbeing via music attitude during quarantine time period?

The remaining sections of this paper are organized as follows: Section 2 provides an overview of the literature review and hypotheses development. Section 3 presents the research methodology, while Section 4 reports the analysis and results. In Section 5, the authors discuss the findings, and Section 6 describes the implications of the research. Section 7 elaborates future research opportunities, and finally, in Section 8, the authors draw the conclusion of the study.

## 2. Literature review and hypotheses development

### 2.1. Social cognitive theory (SCT)

Albert Bandura’s Social Cognitive Theory (SCT) has proven to be considerably useful for researchers studying general human attitude-behavior relationships (Bandura, 1986; Hartley, 2022; Xing and Rojewski, 2022). This study employs SCT to analyze the impact of music attitude precursors on the psychological well-being of immigrants during the COVID-19 quarantine period, incorporating key variables such as cognitive, environmental, and cultural factors, as well as the resulting psychological response. Furthermore, the current study employs SCT as a method for constructing scale items, consistent with recent theoretical advances in music and psychological well-being that increasingly recognize the significance of aims and effects in musical listening experiences (Van Goethem and Sloboda, 2011; Baltazar and Saarikallio, 2016; Schäfer, 2016; Groarke and Hogan, 2018).

While social cognitive theory has been employed in various fields, such as banking (Damayanti et al., 2022), information sharing management (Wang et al., 2021), and economics and administrative sciences (Jader, 2021), there are limited studies linking social cognitive theory to cognitive, environmental, and cultural factors. Groarke and Hogan (2018); Shi et al. (2021) have also discussed the relationship between music experience and well-being in the context of social cognitive theory, which suggests that individuals learn and adopt attitudes, beliefs, and behaviors through observing others and the consequences of their actions (Wang et al., 2021). Onat Kocabiyik (2021) discusses her experience with social media usage during COVID-19 using social cognitive theory.

**Figure 1:**
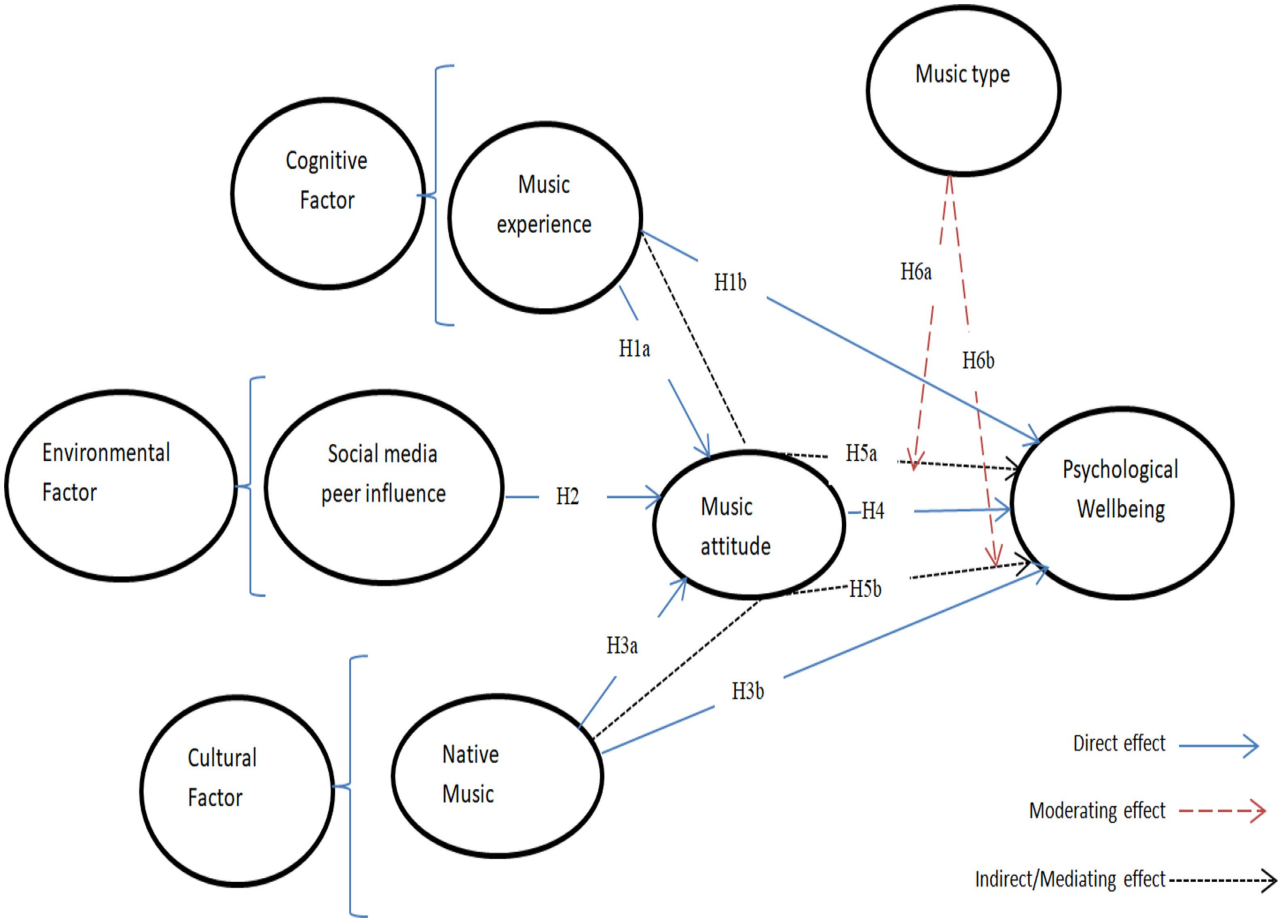
Theoretical research model.

Therefore, based on SCT guidelines, this study explains how musical experience, social media peer influence, and native music stimulate music attitudes, which may further affect the psychological well-being of immigrants during the COVID-19 quarantine period. Figure 1 illustrates the relationships among aforementioned music attitude factors and associated consequences — psychological wellbeing. SCT provides a framework for understanding the complex interplay between music experience, social media peer influence, native music, music attitude, and psychological well-being.

### 2.2. Music experience, music attitude, and psychological wellbeing

Music experience refers to engaging with music for a period of time, and spending more time with music can potentially reduce anxiety and depression, especially during the COVID quarantine. Many people believe that engaging with music has the potential to relieve psychological discomfort, particularly during the pandemic (Loveday et al., 2022). The pandemic has had a significant impact on everyone, causing job loss, financial difficulties, and mental health problems (Spiro et al., 2021). Therefore, it may seem paradoxical to observe such a high prevalence of mental health issues among artists (Resende and de Figueiredo Rocha, 2022). Additionally, Lange and Sun (2022) stated that music can widely disseminate information on the present COVID-19 pandemic, reaching audiences that traditional media such as newspapers, radio, and television may not reach. As a result, music might increase our ability to inform a greater percentage of the population about public health initiatives (Lange and Sun, 2022). Music can be used in strategies aimed at preventing the spread of the coronavirus, in addition to its utility in reducing stress and promoting wellbeing. COVID-19 has greatly affected music-related activities (Kegelaers et al., 2022), and many studies examine the past experience of music (Holkmann-Reid, 2009; Knaggs, 2011; Carter, 2018), musical sensitivity and activity from past music experience (Cartagena, 2021), musical events and related tourist activities based on musical experience (Hiller and Gardstrom, 2018), and the relationship between early music lessons and late-life cognitive functioning (Strong, 2022).

Moreover, the current study reveals that spending more time with music can create a habit, leading to repeated listening, which has the potential to alleviate mental discomfort during the COVID quarantine. People’s engagement with music during the pandemic also influences their mental wellbeing and is influenced by their past music experiences. According to social cognitive theory, this study contributes to a new direction in understanding how music experience as a cognitive factor improves music attitude, which further enhances psychological wellbeing among immigrants during the COVID quarantine. Therefore, we propose:

*H1a*: Music experience (MEX) has a positive, direct and significant effect on immigrants’ music attitude (MA) during Quarantine period.

*H1b*: Music experience (MEX) has a positive, direct, and significant effect on an immigrants’ psychological wellbeing (PW) during Quarantine period.

### 2.3. Social media peer influence relationship with music attitude

According to the study, social media peer influence is an environmental factor that can influence attitudes toward music by sharing or engaging with music via social media. Since social media is a powerful tool for information transfer and sharing in this modern world, it has been used more during the COVID pandemic (Riaz et al., 2021). The study emphasizes that social media peer influence helps individuals develop stronger beliefs during the crucial times of COVID quarantine. Furthermore, people share their quarantine experiences on social media, demonstrating how they defeated the virus and maintained a healthy lifestyle during this difficult period (Vandenberg et al., 2021). Many studies have also highlighted the importance of social media and peer influence during COVID-19 (Dutta et al., 2022; Hamilton et al., 2022; Latikka et al., 2022).

The literature review emphasizes the significance of consistent melodic reactions to cultural emergencies such as lockdowns, which have been found to have exclusively positive effects on psychological well-being (Loveday et al., 2022). Scholars have found that having consistent musical activities can be important for maintaining positive psychological well-being during times of crisis (Whitley et al., 2022). In other words, music can play a positive role in helping people feel better mentally and emotionally during challenging times (Waters et al., 2022). Researchers have also found that the return of music during COVID-19 has led to a clear improvement in moods and long-term mental health in the presence of online audiences (Ziv and Hollander-Shabtai, 2022). Based on the existing literature, social cognitive theory, and social media peer influence, it can be inferred that environmental factors can affect individuals’ music attitudes during the COVID-19 pandemic. It is therefore hypothesized:

*H2*: social media peer influence positively affects immigrants’ music attitude during their quarantine period.

### 2.4. Native music association with music attitude and psychological wellbeing

Native music refers to the traditional music of a country or region that provides immense pleasure to listeners compared to other types of music (Gillam, 2012; Daubney and Fautley, 2021). This is because people inherently understand the lyrics, rhythm, and tune of their native music, consciously or unconsciously. Recent research has revealed that native music, as a cultural factor, affects music attitude, which, in turn, influences the psychological wellbeing of multi-cultural individuals during COVID quarantine. However, very few studies have explored the impact of native music (Aplin et al., 2010).

Du and Leung (2022) found that Miao traditional music gained greater respect because it expressed the positive willingness of the Miao community to learn and preserve their native music. Moreover, music has been shown to produce oxytocin, act as a social intermediary, encourage participation and trust, and advance relational trust and mutual connection (Rosenberg et al., 2021). Chinese opera and folk instrumental music, considered native to Fujian, have not been widely appreciated by students (Zheng, 2022). Nonetheless, these native melodies were live-streamed globally and helped alleviate the sense of isolation during the pandemic. Additionally, songs or music in one’s mother tongue or native language, which often evoke a sense of pride and belonging, can also encourage theory of mind, even among opposing groups (Bodner and Bergman, 2017). In the light of existing literature and the influence of the present study, the following hypotheses are suggested:

*H3a*: Native music (NM) has a positive, direct, and significant effect on immigrants’ music attitude (MA) during the quarantine period.

*H3b*: Native music (NM) has a positive, direct, and significant effect on immigrants’ psychological wellbeing (PW) during the Quarantine period.

### 2.5. The relationship of music attitude and psychological wellbeing

Native music refers to the traditional music of a country or region, which often provides immense pleasure to listeners due to their familiarity with its lyrics, rhythm, and tune (Gillam, 2012; Daubney and Fautley, 2021). A recent study has shown that native music, as a cultural factor, can influence music attitude, which in turn affects the psychological wellbeing of multi-cultural individuals during the COVID quarantine. Despite the significance of native music, there is a lack of research in this area (Aplin et al., 2010).

However, Du and Leung (2022) found that Miao traditional music gained greater respect due to its lyrics in the native language of the Miao community, which expressed their positive willingness to learn and appreciate native music. Moreover, music has been found to produce oxytocin, act as a social intermediary, and encourage participation, trust, and rhythmic activities such as walking, dancing, and ritualistic ceremonies, which promote relational trust and mutual connection (Rosenberg et al., 2021). Zheng (2022) noted that Chinese opera and folk instrumental music, which are native to Fujian, were not frequently listened to by students. However, during the pandemic lockdown, these traditional melodies were live-streamed to a global audience, with some tunes and hymns becoming party anthems that alleviated the sense of isolation and despair. In addition, songs or music in one’s mother tongue or native language can promote in-group favoritism and encourage theory of mind even among opposing groups (Bodner and Bergman, 2017). Therefore, the authors put forward the following hypothesis.

*H4*: Music attitude (MA) has a positive, direct and significant effect on immigrants’ psychological wellbeing (PW) during Quarantine period.

### 2.6. Mediating role of music attitude

An optimistic attitude toward musical activities can hold individuals’ attention by stimulating and utilizing different parts of the brain (Krout, 2007). Those with a positive music attitude may adapt to and respond to their talents accordingly (Miranda, 2022). This is why several scholars have considered music and musical activities as great memory boosters that effectively support the understanding of complex tasks (Särkämö et al., 2014; Damsgaard and Jensen, 2021; Wang, 2022). There is a growing body of evidence that suggests positive and optimistic thinking about music improves cognitive abilities, which are linked to individuals’ musical experiences (Damsgaard and Jensen, 2021; Schubert, 2022; Wang, 2022). Moreover, spending more time with music may enhance one’s music experience and expertise, resulting in an improvement in music attitude that can promote mental acuity and relaxation (Reybrouck et al., 2022). These scholars also claim that coping mechanisms have become an essential aspect of musicality, which comprises several other cognitive skills such as the capacity to synchronize movements and identify musical pitch. These skills can contribute to the musical experience, which in turn can influence an individual’s music attitude and psychological state of mind.

Music attitude based on music experience is not the only essential relationship that has been considered, but there is also a strong and influential relationship between music attitudes and psychological well-being, particularly among youth (Damsgaard and Jensen, 2021; Greenberg et al., 2022).

Apart from the cognitive factor of music experience (MEX), an individual’s cultural factors such as language, norms, traditions, caste, belief systems, transitions, and native music can influence their music attitude, which may further affect their psychological well-being in daily life. Generally, music is presented through a musical language, and a person cannot enjoy or comprehend it if they do not understand its fundamentals (Shrestha, 2018; Dvorak and Hernandez-Ruiz, 2019). Only those with a foundational understanding of music can appreciate and comprehend it, eventually leading to the esthetic aim of amusement, which can promote mental freshness (Zheng, 2022). Scholars have concluded that to achieve the goal of music education, the content of college public music education should begin with tutoring native music as the foundation of music knowledge in the native language (Gooding and Springer, 2020; Daubney and Fautley, 2021). Due to such a strong relationship of MEX and NM with MA and PW, the authors of this study antedate the following mediation hypotheses.

*H5a*: Immigrants’ Music attitude (MA) positively mediates the relationship between music experience (MEX) and psychological wellbeing (PW) during Quarantine period.

*H5b*: Immigrants’ Music attitude (MA) positively mediates the relationship between native music (NM) and psychological wellbeing (PW) during Quarantine period.

### 2.7. Moderated mediation role music type

Music is one of the most ubiquitous forms of human expression, pervading the daily lives of people from diverse backgrounds and worldwide (Angel-Alvarado et al., 2022). Numerous studies, both observed and experimental, have demonstrated the widespread benefits of music and its influence on various aspects of human existence, including physical, sociocultural, academic, psychological, and emotional well-being (Särkämö et al., 2014; Rose et al., 2019; Whiteman, 2020; Oliver et al., 2021).

Music has also been utilized in prior research to investigate individuals’ preferences and behaviors (Kim and Kang, 2021; Greenberg et al., 2022); as well as its therapeutic effects on mental health and other ailments (Ysseldyk et al., 2021). Studies focusing on music and its types have shown potential for reducing stress and improving well-being in youngsters, particularly in young students (Saarikallio et al., 2020). Young people have reported that listening to a diverse range of music can help them relax, manage their emotions, and cope with difficulties (Kim and Kang, 2021).

Moreover, musical engagement is a powerful tool for health and well-being that can benefit people of all ages, backgrounds, and situations. The type of music also plays a moderating role in the relationship between native music preferences and psychological well-being (Flannery and Woolhouse, 2021; Greenberg et al., 2022; Miranda, 2022). Cultural musicology has proposed and provided evidence for music’s ability to promote compassion and social understanding through strong emotional, mental, and social elements (Musgrave, 2022; Li Qiu and Hirunrux, 2022). Despite the caution expressed by musicology about essentializing and glamorizing claims, music’s potential as a “universal language” that transcends cultural differences and promotes social coordination, reverence, and celebration remains prominent (Fanari et al., 2022).

Several researchers have investigated whether people who are better at understanding and feeling other people’s emotions are also better at understanding and feeling emotions expressed through music (Juslin and Laukka, 2004; Hays and Minichiello, 2005; Fink et al., 2021). Furthermore, music and its types have a significant impact on listeners’ physiological well-being. A Finnish study Saarikallio et al. (2020) examined how listening to music affects teens’ reported feelings of autonomy and emotional health, as well as the role of context and individuality. Based upon the above discussion, the authors have put forward the following hypotheses:

*H6a*: Music type (MT) positively moderates the mediating relationship between music experience (MEX) and psychological wellbeing (PW) through immigrants’ music attitude during COVID quarantine period.

*H6b*: Music type (MT) positively moderates the mediating relationship between native music (NM) and psychological wellbeing (PW) via immigrants’ music attitude during COVID quarantine period.

## 3. Methodology

### 3.1. Questionnaire design

The questionnaire items have been adapted from highly cited research articles that have validated survey instruments. Some questionnaire items were slightly altered.

#### 3.1.1. Independent variables

The set independent variables are related to cognitive, environmental and cultural factors. Among them music experience is related to cognitive factors. The items for Music Experience (MEX) were chosen from Du and Leung (2022), Strong (2022), and Völker (2022). This instrument comprises 4 items that measure the individuals’ past experience with music, especially their thinking toward music during the COVID quarantine. Furthermore, MEX examines how motivation or attitude toward music affects an individual’s psychological well-being during the quarantine period in China.

Second social peer influence is related to environmental factors. Social media peer influence (SPI) has been evaluated by drawing on the items from Järvekülg and Wikstroem (2022). This instrument contains 4 reflective items that entail the individual’s social media connections and relationships with his friends, family members, and other preference groups, and how their influence may affect his attitude music and his mental peace during COVID quarantine.

Third, native music is related to cultural factors. Native music (NM) was assessed by the items that were self-developed by the author. Four reflective items can reflect the outcomes that may attract an individual, intentionally or unintentionally, to listening to cultural music in his or her native language in quarantine centers. Besides, NM entails whether the motivation toward native music will bring forth an individual’s psychological wellbeing or not.

#### 3.1.2. Mediating variable

The authors of this study have chosen music attitude as mediating variable. The items for music attitude (MA) were adapted from Lalchuangkima (2022) and slightly modified to align with the study objective. Four items measure the immigrant’s musical motivations, which may further affect his or her mental wellbeing during the quarantine period in China.

#### 3.1.3. Moderating variable

Music type has been taken as a moderating variable in this research. The items for music type (MT) were taken from Kim and Kang (2021) and Greenberg et al. (2022), and the items have been adapted according to the requirements of this research. Four items entail whether music varieties may influence an individual’s music motivations and his psychological peacefulness, particularly during the quarantine period in China.

#### 3.1.4. Dependent variable

Finally, in this study psychological wellbeing has been taken as dependent variable, the items for psychological wellbeing (PW) were adopted from Loveday et al. (2022) and Pheil (2022). There are five items used to assess individuals’ mental well-being as a result of music attitude and its precursors during the COVID quarantine period.

All of the items were assessed on a seven-point Likert scale that ranged from 1 = strongly disagree, 2 = disagree, 3 = slightly disagree, 4 = neither agree nor disagree, 5 = slightly agree, 6 = agree, 7 = strongly agree. The reason to use 7 Point Likert scale is the most accurate type among other scales to gather and analyze Likert scale data (Tarka, 2017; Taherdoost, 2019). It gives a better image of the respondent’s true opinion. To confirm a general reliability check, the questionnaire items and instruments were cross-checked by five PhD students related to music behavior. Subsequently, two professors reviewed the questionnaire for further corrections and authentication. Following that, for the convenience of the study sample, i.e., immigrants from different countries, the questionnaire was prefer to designed in English. Because questionnaires are often presented in English due to its wide acceptance as a language of international communication and its perceived neutrality. As English is often used as a common language for business, science, and academia, as well as for many international organizations.

### 3.2. Research settings and data collection

The study population included immigrants (from various countries) who traveled to China and were quarantined in the country’s five different isolation centers during the COVID pandemic, especially during the transmission of Omicron-led COVID 4th wave. Keeping in mind the convenience of respondents and the need to protect against the COVID 4th wave-Omicron transmission, data for this study were collected using a survey method by online questionnaires in English language as per respondents (immigrants) convenience from diverse countries. To understand the impact of music attitude precursors and their influence on immigrants’ psychological wellbeing during strict quarantine period after entering China. This is because, it has been identified that during COVID pandemic people with more stress-level tended to use music more purposefully for their psychological well-being (Terasawa et al., 2021). The survey spanned 45 days from April 15th, 2022, to June 5th, 2022. The data were gathered from the immigrants who entered China during pandemic, since they were gone through a strict and prolonged quarantine period on their arrival to China and they were considered as the best suitable research sample to proceed this study. To validate the conceptual model and the hypotheses discussed in the previous section, the authors reviewed previous quantitative studies and prepared a questionnaire identifying the constructs from literature. To check if the questions genuinely represent the measures of all constructs and that the respondents are able to respond to the measures meaningfully, a pilot study has been conducted to check the statistical reliability in the form of internal consistency of the constructs and the items. By following, Hinkin (1998) suggestion to ensure the reliability and validity of the chosen items, the authors conducted a pilot study. The author gathered data from first 40 immigrants who entered China and were quarantined during the last COVID wave. Initial study found a range of 0.744–0.924 for the internal consistency of the variables, which satisfies Hair et al.’s (2014a) minimal value requirement of 0.7. The pilot and main survey of the study were conducted as early as possible, and a minimal time gap was maintained so that it did not create any impact on the study. The authors of this study divided the questionnaire into two sections: the first section contained demographic questions related to immigrants’ personal and professional information, and the second section consisted of items based on the construct framed in the proposed conceptual model. The questionnaire dictated the context and purpose of this study. The final questionnaire consisted of 25 items from six constructs with seven demographic questions. For data collection, convenience sampling methods were considered. For the online survey, immigrants were contacted through WeChat, Facebook, and WhatsApp etc. The study was quick in terms of collecting surveys because social media platforms were used, and most young respondents prominently use above mentioned social media platforms on daily routine bases (Baltar and Brunet, 2012). In addition, immigrants who responded during data collection process were requested to circulate the questionnaire among their colleagues who were quarantined. Finally, out of 520 circulated questionnaires the authors received 350 responses from the respondents, out of which 50 were discarded because of missing responses and recursive answers. Hence, 300 were retained as valid and correct responses resulting in a validity percentage of 57.69%. The authors have present a brief description of demographics in Table 1. Adequate sample size; that is, 10 times the number of items, was considered (Tabachnick and Fidell, 2007).

**Table 1:** Demographic of respondent (*N* = 300).

Characteristics		Frequency proportion	Proportion%
Gender	Female	130	43.33
	Male	170	56.66
Age	20 to 25 years	29	9.66
	26 to 30 years	48	16
	31 to 35 years	97	32.33
	36 to 40 years	88	29.33
	41 and above	38	12.66
Education	PhD	87	29
	Diploma student	20	6.66
	Masters	58	19.33
	Bachelors	20	6.66
	Post Doc	115	36.66
Quarantine center	Wuhan	74	24.66
	Zhengzhou	61	20.33
	Beijing	90	30
	Guangzhou	40	13.33
	Xian	35	11.66
Occupation	Student	25	8.33
	University Researcher	98	32.66
	Teacher	39	13.00
	Diplomat	51	17
	Businessmen	87	29
Nationality	Russian	67	22.33
	Korean	39	13
	Pakistani	64	21.33
	Arabians	26	7.66
	African	46	15.33
	Any other country	58	19
During quarantine, mostly I prefer to enjoy music from	Internet	128	42.66
	Mobile	68	22.66
	Laptop	50	16.66
	TV	15	5
	Any other	39	13

### 3.3. Multicollinearity test

Before conducting SEM analysis, it is crucial to examine the presence of multicollinearity constraints. Multicollinearity occurs when two or more exogenous variables have a high correlation (Abraham et al., 2021). To evaluate multicollinearity, two commonly used measures are the variance inflation factor (VIF) and tolerance (Hair et al., 2014b). The VIF is the primary measure used to test multicollinearity. If the VIF value is less than 3.0, it is assumed that there is no issue with multicollinearity (Bhukya and Singh, 2015). In the current study, the VIF values ranged from 1.768 to 2.232, which is Bhukya and Singh (2015) within the acceptable range of 3.0. The second measure, tolerance, ranges from 0.1 to 1.0 according to Kutner et al. (2004). The tolerance values in this study were all within the permissible range, ranging from 0.450 to 0.560. Based on these results, it can be concluded that this research does not have any issues with multicollinearity.

## 4. Data analysis

In order to undertake an effective data analysis method, first, the authors conducted a demographic and descriptive teste of the collected data. Second, the entire model fitness was calculated using a variety of statistical methods, e.g., chi-square, comparative fit index (CFI), root mean square error of approximation (RMSEA), and Tucker–Lewis index (TLI). Thirdly, confirmatory factor analysis (CFA) was used to verify and validate the measurement model. Finally, a structural equation model (SEM) was used to examine the constructs’ relationships. The authors utilized SPSS 24 and AMOS 24 to conduct all of the statistical tests stated above.

### 4.1. Demographic analysis

Table 1 presents the respondents’ demographic information. The majority of the respondents were male (170, 56.66%). The respondents’ ages between 31 to 35 years are greater in number (97, 32.33%). In terms of educational level, 115 (36%) respondents are pursuing post-doctorate (during the period of data collection, many Chinese universities permitted post-doctoral fellows to enter China due to COVID-19 restrictions as compare to Bachelor’s, Master’s, and PhD students). While the rest have completed diploma studies, bachelor’s degrees, master’s degrees, and PhD degrees, respectively. In terms of the number of immigrants in quarantine centers, the Beijing quarantine center has a higher number, 90, or 30%, as compared to all quarantine centers combined. Among the total respondents, 98 (32.66%) are university researchers, and are larger in number, while the rest are comprised of teachers, students, diplomats, and businessmen. In terms of nationality, more Russian immigrants travel to China (67, 22.33%), followed by Pakistanis (64, 21.33%), Africans (46, 15.33%), Koreans (39, 13%), and others (58, 19%). Finally, among all, 128 respondents (42.66%) have used the internet to enjoy music during their isolation periods in different quarantine centers, respectively.

### 4.2. Descriptive statistics

Table 2 presents the descriptive statistics of all variables that were examined in the study. The skewness and kurtosis values included in the table indicate the normality of the data, which is a necessary condition for conducting SEM analysis.

**Table 2:** Descriptive statistics of all constructs.

Construct	Nationality	Mean	Range	S.D	Skewness	Kurtosis
MEX	Russian	4.90	5.25	1.40	−1.40	1.28
	Pakistani	5.25	4.50	1.06	−1.42	2.00
	African	5.63	4.25	1.01	−1.34	1.47
	Korean	5.30	5.75	1.10	−1.24	1.97
	Arabian	5.21	6.00	1.18	−1.46	2.0
	Any other country	5.22	5.00	1.15	−1.21	1.13
SPI	Russian	5.28	4.67	1.19	−1.46	1.89
	Pakistani	5.30	4.67	0.93	−1.52	2.14
	African	5.60	4.00	0.96	−0.93	0.21
	Korean	5.11	5.67	1.45	−1.15	0.68
	Arabian	5.44	5.00	1.07	−1.06	1.14
	Any other country	5.31	4.67	0.92	−1.15	2.47
NM	Russian	5.62	4.33	1.17	−1.34	1.59
	Pakistani	5.45	5.60	1.40	−1.10	0.60
	African	5.78	5.00	1.06	−1.50	2.05
	Korean	5.26	5.67	1.22	−1.21	1.76
	Arabian	5.41	6.00	1.26	−1.25	1.61
	Any other country	5.36	4.67	1.07	−0.96	0.26
MT	Russian	5.71	5.67	1.01	−1.43	2.07
	Pakistani	5.58	4.33	1.03	−1.23	1.53
	African	5.94	5.33	1.03	−1.38	1.99
	Korean	5.57	6.00	1.30	−1.14	1.48
	Arabian	5.69	5.67	1.20	−1.21	1.38
	Any other country	5.56	4.00	1.07	−0.84	0.82
MA	Russian	5.20	4.75	1.07	−1.06	2.01
	Pakistani	5.24	4.50	1.02	−0.87	0.84
	African	5.61	6.00	1.06	−1.16	0.90
	Korean	5.45	5.75	1.21	−1.32	1.33
	Arabian	5.76	5.00	1.08	−1.24	2.10
	Any other country	5.46	4.50	0.87	−0.94	1.27
PW	Russian	5.77	4.25	1.01	−1.47	2.16
	Pakistani	5.93	4.80	0.98	−0.89	1.54
	African	5.87	6.00	1.07	−1.12	1.19
	Korean	5.49	5.75	1.20	−1.23	1.77
	Arabian	5.26	5.96	1.11	−1.32	1.85
	Any other country	5.34	4.25	0.85	−1.18	2.10

### 4.3. Measurement model

The measurement model was evaluated by calculating the overall model fit, as illustrated in Table 3, construct reliability test, and validity test. The values of measurement model fit indices are as follow:

**Table 3:** Fit indices of the measurement model.

Model fit index	Acceptable levels	Obtained fit estimates	Interpretation
*x^2^*	---	267.856	----
*df*	---	174	----
P	<0.05	000	Excellent
*x^2^/df*	<5 (Bentler, 1989)	1.539	Excellent
CFI	>0.90 (Hu and Bentler, 1999)	0.972	Better
RMSEA	<0.08 (MacCallum et al., 1999)	0.042	Excellent
TLI	>0.8 (Hooper et al., 2008)	0.967	Excellent

x^2^:(CMIN), x^2^/df; Minimum discrepancy, CFI, Comparative Fit Index; RMSEA, Root mean square error of approximation; TLI, Tucker Lewis index.

Reliability tests were conducted using composite reliability (CR) and Cronbach’s alpha. The values of CR and Cronbach’s alpha must be equal to cut off score i.e. 0.70 or above Carter et al. (2009) for each variable. CR gives the construct internal consistency and is reflected as an appropriate substitute for Cronbach’s alpha (Hair et al., 2010). In this research, the above-mentioned conditions for reliability tests were fulfilled. The Cronbach’s alpha ranged from 0.800 to 0.879 and the CRs from 0.806 to 0.879 (see Table 4). Therefore, all the variables in this study have met the cut off score and are accepted.

**Table 4:** Confirmatory factor analysis.

Constructs & items	Factor loading	SMC	CR	Cronbach’s α	AVE
Music experience (MEX)			0.879	0.878	0.645
MEX1	0.79	0.62			
MEX2	0.81	0.65			
MEX3	0.76	0.58			
MEX4	0.85	0.73			
Social media peer influence (SPI)			0.828	0.825	0.616
SPI2	0.84	0.71			
SPI 3	0.82	0.67			
SPI4	0.86	0.75			
Native music (NM)			0.88	0.879	0.708
NM2	0.76	0.58			
NM3	0.83	0.69			
NM4	0.76	0.58			
Music type (MT)			0.824	0.827	0.610
MT2	0.80	0.64			
MT3	0.82	0.66			
MT4	0.72	0.52			
Music attitude (MA)			0.848	0.839	0.585
MA1	0.76	0.58			
MA2	0.83	0.68			
MA3	0.80	0.64			
MA4	0.66	0.43			
Psychological wellbeing (PW)			0.806	0.796	0.509
PW1	0.74	0.54			
PW2	0.74	0.55			
PW3	0.71	0.51			
PW4	0.66	0.43			

CR, Composite Reliability; SMC, Square Multiple Correlation; AVE, Average Variance Extracted.

**Figure 2:**
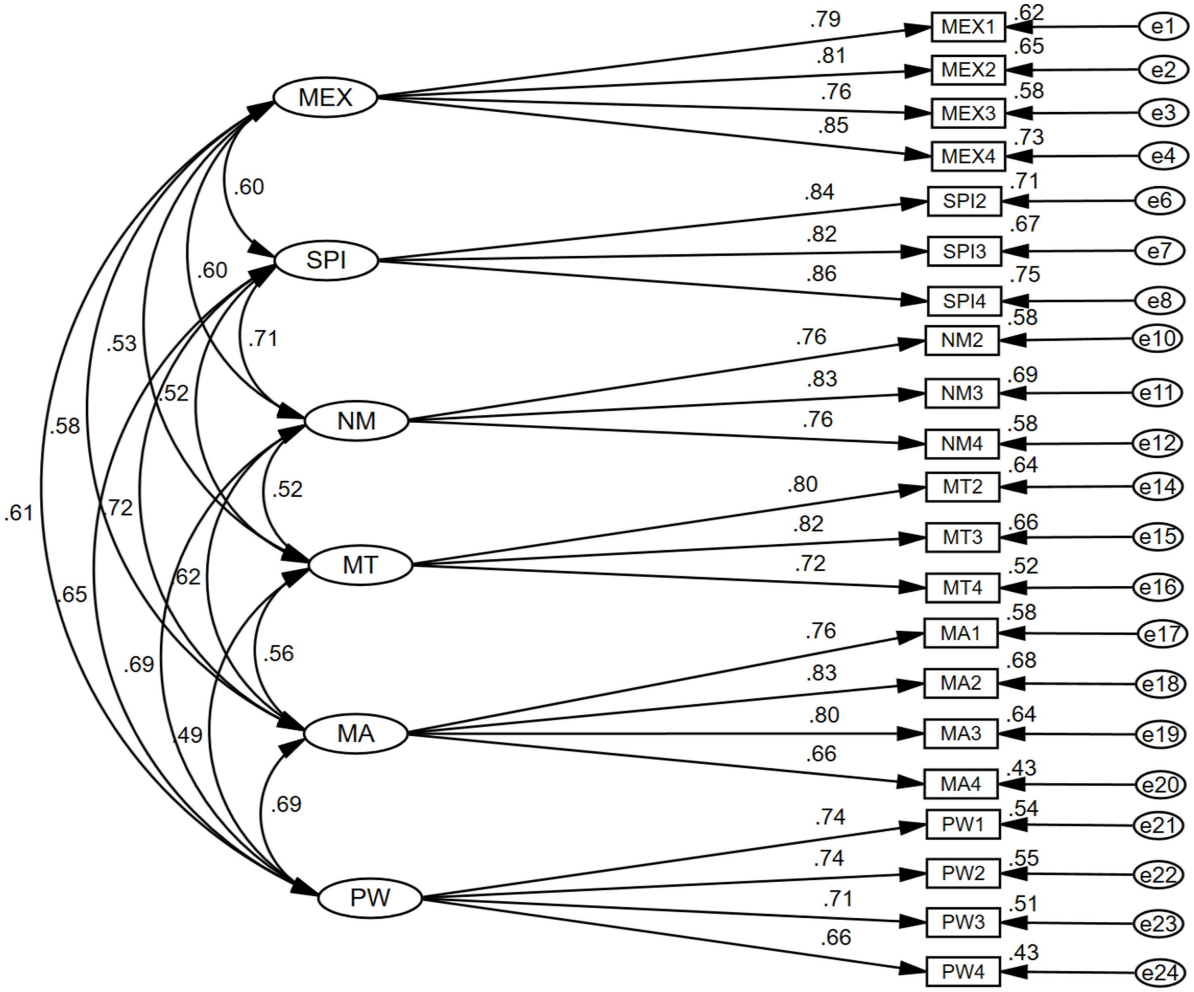
Measurement model/CFA.

The discriminant validity and construct validity of the model were also evaluated. All the items and constructs’ (variables) cut off score for construct validity assessments are: (1) all the factor loadings must be greater than or equal to 0.60 (Henseler et al., 2015). (2) The value of CR for each construct must be at least 0.70 or above (Chin, 1998; Quiroga-Garza et al., 2021). (3) The average variance extracted (AVE) value should be at least 0.50 (Fornell and Larcker, 1981). After removing items (SPI1, NM1, MT1, and PW5) that caused problems when calculating AVE due to low factor loadings, all other remaining items and constructs (study variables) met the convergent validity cut off score. The CR values of all items range from 0.806 to 0.879; the AVE values range from 0.509 to 0.708; and all factor loading values range from 0.66 to 0.86, showing good convergent validity. For a decent square multiple correlation (SMC), the value of all items must be greater than or equal to 0.36, because SMC articulates how well an item measures a construct (Hair et al., 2010; Henseler et al., 2015). In terms of SMC this study has values ranging from 0.43 to 0.75 (See Table 4; Figure 2).

### 4.4. Discriminant validity analysis

To obtain a suitable discriminant validity, the square root of the average variance extracted (AVE) must be greater than the corresponding correlations between that construct and the residual of other constructs (Chin, 1998). In order to get reasonable discriminant validity results, we compared the square root of the average variance extracted from every construct and its correlation coefficient with other constructs (see Table 5).

**Table 5:** Discriminant validity.

	CR	AVE	MEX	SPI	NM	MT	MA	PW
MEX	0.879	0.645	**0.803**					
SPI	0.879	0.708	0.596***	**0.842**				
NM	0.828	0.616	0.600***	0.706***	**0.785**			
MT	0.824	0.610	0.530***	0.523***	0.516***	**0.781**		
MA	0.848	0.585	0.583***	0.723***	0.615***	0.563***	**0.765**	
PW	0.806	0.509	0.610	0.651^†^	0.688	0.488	0.688	**0.714**

MEX, music experience; SPI, social media peer influence; NM, native music; MT, music type; MA, music attitude; PW, psychological wellbeing. Significance threshold values: ^†^*p* < 0.100, *p < 0.050, **p < 0.010, ***p < 0.001. It just shows that the bold value should need to be the greater than the prior values horizontally and following values down vertically.

### 4.5. Structural model

**Figure 3:**
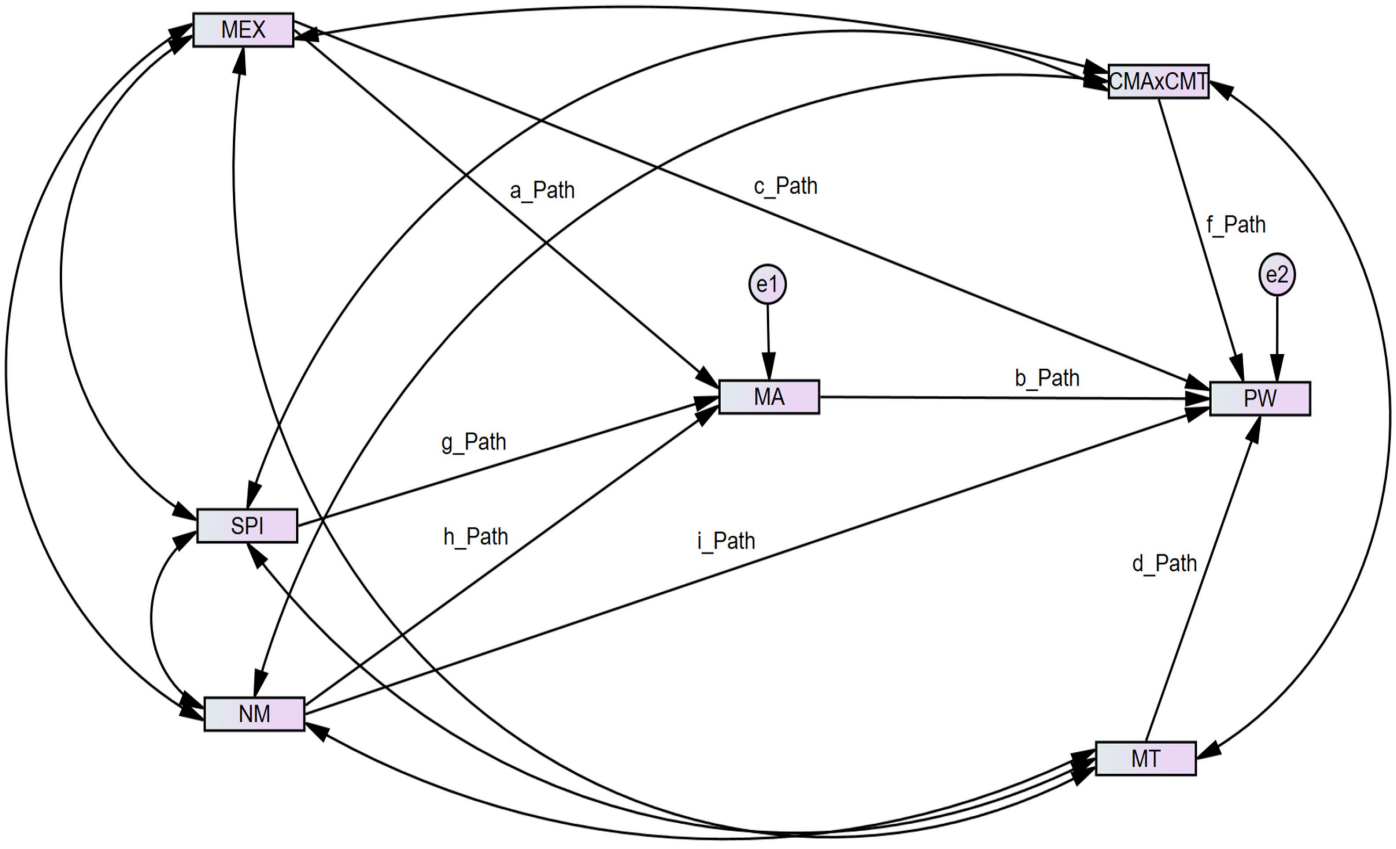
Model along with parameters for SEM and moderated mediation analysis. MEX, music experience; MT, music type; SPI, social media influence; NM, native music; MA, music attitude; CMA × CMT, interaction term (for moderated mediation); PW, psychological wellbeing.

Prior to perform structural equation model the authors have examined overall model fitness of structural model. Table 6 presents structural model fit indexes as follow. To perform structural equation modeling along with mediated moderation analysis via AMOS 24, the author has named parameters (paths) as shown in Figure 3 to construct user-define estimands that will be helpful to perform SEM analysis, in particular to examine the moderated mediation model.

**Table 6:** Fit indices of the structural model.

Model fit index	Acceptable levels	Obtained fit estimates	Interpretation
*x^2^*	---	11.715	----
*df*	---	4	----
P	<0.05	0.02	Excellent
*x^2^/df*	<5 (Bentler, 1989)	2.92	Excellent
CFI	>0.90 (Hu and Bentler, 1999)	0.99	Better
RMSEA	<0.08 (MacCallum et al., 1999)	0.070	Excellent
TLI	>0.8 (Hooper et al., 2008)	0.94	Excellent

x^2^:(CMIN), x^2^/df; Minimum discrepancy, CFI, comparative fit index; RMSEA, root mean square error of approximation; TLI, Tucker Lewis index.

Based on Cheah et al. (2021), the authors of this study have conducted SEM-based moderated mediation analysis via AMOS 24. Therefore, the following formulas have been used while defining user estimates: The paths that have been used to construct user-defined estimates have been mentioned in Figure 3 and Table 7.

**Table 7:** Formula based on Figure 3 parameters/user define estimands.

1. IndirectAB = a_Path*b_Path
OneStandBelowAB = a_Path*((f_Path*-2.05706) + b_Path)
OneStandHighAB = a_Path*((f_Path*2.05706) + b_Path)
IOMM_1 = f_Path*a_Path
2. IndirectHB = h_Path*b_Path
OneStandBelowHB = h_Path*((f_Path*-2.05706) + b_Path)
OneStandHighHB = (h_Path+(f_Path*2.05706) + b_Path)
IOMM_2 = f_Path*h_Path

2.05706: Standard Deviation of interaction term (CMA × CMT); IOMM, Index of Moderated Mediation.

**Figure 4:**
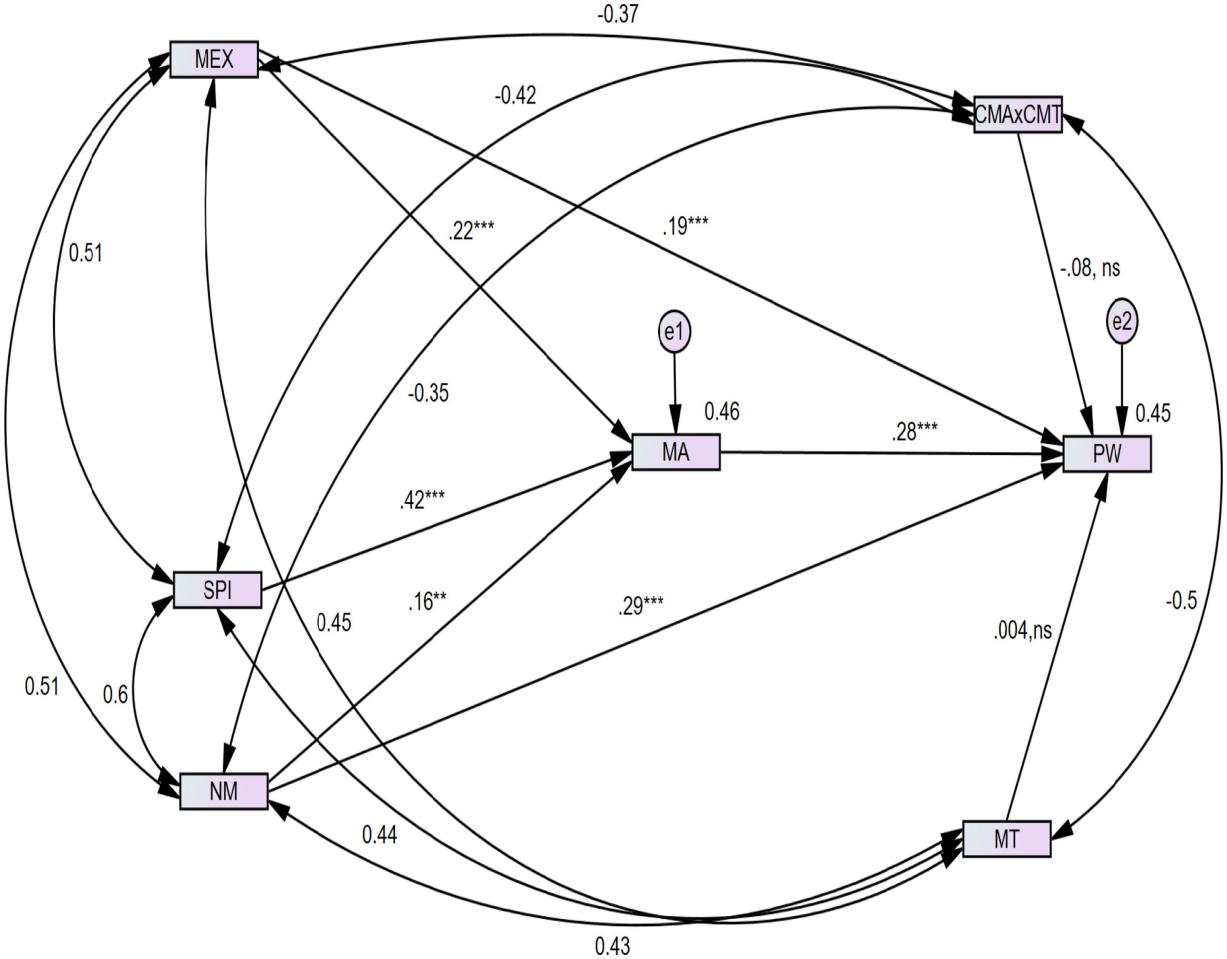
Structural model results. MEX, music experience; CMA × CMT, interaction term (for moderated mediation); MT, music type; SPI, social media influence; NM, native music; MA, music attitude; PW, psychological wellbeing. Significance threshold values: ^†^*p* < 0.100, *p < 0.050, **p < 0.010, ***p < 0.001, ns, not significant.

The results of the structural equation model (SEM) test are presented in Figure 4. According to the research model, all direct and indirect hypotheses are significant, but the moderated mediation hypotheses are statistically not significant. Details are as follows:

*Direct relationships:* MEX has a significant and positive effect on MA (*β* = 0.22***, *p* < 0.001), hence, supporting H1a. MEX has a direct, supportive, and positive effect on PW (*β* = 0.19***, *p* < 0.001), indicating that H1b is significant and positive. H2, which corresponds SPI is positively and significantly effects on MA (*β* = 0.42***, *p* < 0.001). Similarly, H3a which denotes NM, has a significant, positive, and direct effect on MA (*β* = 0.16**, *p* < 0.01). H3b which corresponds to NM, has a positive, significant, and direct effect on PW (*β* = 0.29***, *p* < 0.001). In addition, according to H4, MA is positively, significantly, and directly influencing PW (*β* = 0.28***, *p* < 0.001) (Table 8).

**Table 8:** Direct hypotheses testing.

Hypotheses	Relationship	C.R. (*t* value)	*p*	Standardized structural coefficients (*β*)	Interpretation
H1a	MEX → MA	4.281	0.001***	0.22***	Significant
H1b	MEX → PW	3.502	0.001***	0.19***	Significant
H2	SPI → MA	7.553	0.001***	0.42***	Significant
H3a	NM → MA	2.943	0.003**	0.16**	Significant
H3b	NM → PW	5.308	0.001***	0.29***	Significant
H4	MA → PW	5.283	0.001***	0.28***	Significant

MEX, Music Experience; SPI, Social Media Peer Influence; NM, Native Music; MA, Music Attitude; PW, Psychological Wellbeing; ns, Not Significant. Significance threshold values: ^†^*p* < 0.100, *p < 0.050, **p < 0.010, ***p < 0.001.

**Figure 5:**
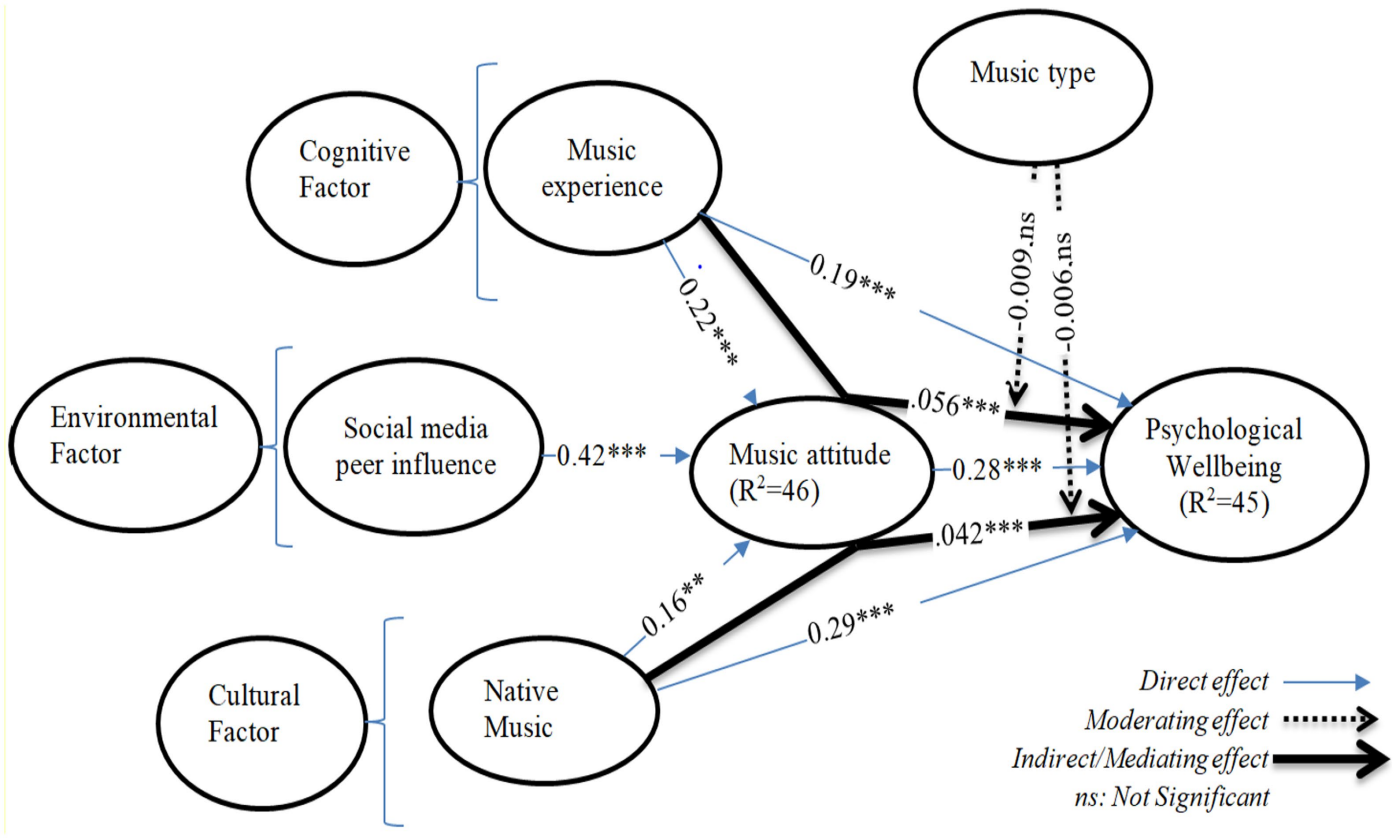
Structural model with direct, mediating, and moderated mediation hypotheses Significance threshold values: ^†^*p* < 0.100, *p < 0.050, **p < 0.010, ***p < 0.001.

*Mediating/Indirect relationship:* The authors of this study tested the indirect/meditation effect using Baron and Kenny (1986) recommendations. To conduct an indirect/mediation analysis, the author has used the bootstrapping tool by fixing a 95% confidence interval (CI) and 5,000 bootstrap samples. The results are shown in Table 9 and Figure 4, which report that all the mediation conditions were fulfilled and satisfied according to Baron and Kenny (1986) point of view (Figure 5).

**Table 9:** Indirect/Mediating hypotheses testing.

Hypothesis	Relationship	Direct effect	Indirect effect	Confidence intervals		*p* value	Interpretation
				Lower Bounds (BC)	Upper Bounds (BC)		
H5a	MEX → MA → PW	0.19	0.056	0.021	0.130	0.001***	Partial mediation
H5b	NM → MA → PW	0.29	0.042	0.013	0.105	0.002**	Partial mediation

MEX, Music Experience; NM, Native Music; MA, Music Attitude; PW, Psychological Wellbeing. Significance threshold values: ^†^*p* < 0.100, *p < 0.050, **p < 0.010, ***p < 0.001.

In the presence of MA (the mediator), MEX (the independent variable) correlated and mediated the relationship with PW (the dependent variable) (*β* = 0.056***, *p* < 0.001). As soon in Figure 5 in addition, there is no zero between the values of the lower bound (BC) (0.021) and the upper bound (BC) (0.130). As a result, H5a exhibits a partial mediation relationship. Because, the direct relationship between MEX and PW is also significant and supportive, i.e. (*β* = 0. 0.19***, *p* < 0.001). Hence, the author has declared that H5a presents a partial mediation relationship between MEX and PW through MA. Furthermore, the author found that the relationship between NM and PW has also been mediated, i.e. (*β* = 0.042**, *p* < 0.002) due to the existence of MA as a mediator between the dependent and independent variables. Besides, there is no zero between the values of the lower bound (BC) (0.013) and the upper bound (BC) (0.105). Hence, the author found that H5b is significant and brings forth partial mediation between the independent variable (NM) and dependent variable (PW) via the mediating variable (MA), because the direct effect between NM and PW is also significant and supportive, i.e. (*β* = 0.29***, *p* < 0.001). Hence, it is proved that all the indirect or mediating hypotheses are positive and significant.

*Moderated mediation relationships*: Finally, we examine the moderated effect of music type (MT) on the mediating relationship of music experience (MEX) and psychological wellbeing (PW) via music attitude (MA) and on the mediating relationship of native music (NM) and psychological wellbeing (PW) through music attitude (MA). as shown in Table 10 and Figure 4, Results of the moderated mediation analyses via AMOS SEM for hypothesis testing can be seen Table 10 shows the results of the moderated mediation effects at low and high levels of music type. As the indirect effect between MEX and PW via MA is significant, i.e., the estimate is 0.056, having 95% CI = [Lower = 0,021, upper = 0.130] and having *p* value 0.001. In addition, the values of 1 standard deviation below are 0.074 estimates, [lower = 0.029, upper = 0.155] with a *p* value of 0.001, which is significant. As for as 1 standard deviation above the estimate is 0.039, [Lower = 0.010, upper = 0.117] with a significant p value which is 0.006. Furthermore, while examining the indirect effect between NM and PW via MA the author found that the estimate = 0.042, 95% CI = [Lower = 0.013, upper = 0.105] along with a *p* value = 0.002 which is significant. Besides, on calculating 1 standard deviation below the mean, the estimate is 0.055, CI 95% = [lower = 0.018, upper = 0.118], and the *p* value = 0,003 which is significant. As for as 1 standard deviation above the mean, estimates = 0.130, CI 95% = [Lower = 0.008, upper = 0.244], *p* value 0.040 which is significant. To further test the moderated mediation effects, this study calculated the indexes of moderated mediation separately. The results reveal that the mediating relationship between MEX and PW via MA has not been significantly moderated by music type (MT). As the index of moderated mediation (IOMM_1) = −0.009, 95% CI = [lower = −0.028, upper = 0.007], and the *p* value is 0.241, i.e., statistically not significant. Therefore, the author claimed that there is no moderated mediation effect of MT on the indirect relationship between MEX and PW through MA. Hence, H6a is statistically not significant. In addition to examine the moderated mediation effect of MT on the indirect relationship between NM and PW via MA, the authors calculated index of moderated mediation (IOMM_2) = − 0.006, 95% CI = [lower −0.021, upper = 0.044], *p* value is 0.187, i.e., statistically not significant. Therefore, the author has confirmed that H6b is statistically not supported (Tables 10 and 11).

**Table 10:** Moderated mediation analysis.

Direct relationships	Unstandardized estimates	Standardized estimates	CR (*t* values)	*p* values
MEX → MA	0.21	0.22***	4.281	0.001***
MEX → PW	0.17	0.19***	3.502	0.001***
SPI → MA	0.40	0.42***	7.553	0.001***
NM → MA	0.15	0.16**	2.943	0.003**
NM → PW	0.26	0.29***	5.308	0.001***
MA → PW	0.27	0.28***	5.283	0.001***
MT → PW	0.003	0.00	0.071	0.944. ns
CMA × CMT → PW	−0.042	−0.08	−1.637	0.102, ns

Indirect relationships	Direct effect	Indirect effect	CI = [Lower, Upper]	*p* value
MEX → MA → PW	0.19***	0.056***	[0.021, 0.130]	0.001
NM → MA → PW	0.29***	0.042**	[0.013, 0.105]	0.002
Moderated mediation relationships				
At 1 (STD) low level of MT the effect on MEX → MA → PW is		0.074***	[0.029,0.155]	0.001
At 1 (STD) high level of MT the effect on MEX → MA → PW is		0.039**	[0.010, 117]	0.006
Index of Moderated Mediation for Hypothesis 6a (IOMM_1)		−0.009	[−0.028, 0.007]	0.241, ns
At 1 (STD) low level of MT the effect on NM → MA → PW is		0.055**	[0.018,0.118]	0.003
At 1 (STD) high level of MT the effect on NM → MA → PW is		0.341*	[0.073, 0.654]	0.012
Index of moderated mediation for Hypothesis 6b (IOMM_2)		−0.006	[−0.021,0.044]	0.187, ns

CI, Confidence interval; MEX, Music Experience; SPI, Social Media Peer Influence; NM, Native Music; MT, Music Type; CMA × CMT, Interaction term (For moderated mediation); MA, Music Attitude; PW, Psychological Wellbeing; STD, Standard deviation; ns, Not Significant. Significance threshold values: ^†^*p* < 0.100, *p < 0.050, **p < 0.010, ***p < 0.001 (Gaskin et al., 2019).

**Table 11:** Overall hypotheses results.

Hypotheses	*p* values	Status
*Hypothesis 1a*: Music experience (MEX) has a positive, direct and significant effect on immigrant music attitude (MA) during Quarantine period	0.001***	Significant
*Hypothesis 1b*: Music experience (MEX) has a positive, direct and significant effect on immigrants psychological wellbeing (PW) during Quarantine period	0.001***	Significant
*Hypothesis 2*: Social media peer influence (SPI) has a positive, direct and significant effect on immigrants’ music attitude (MA) during COVID quarantine.	0.001***	Significant
*Hypothesis 3a*: Native music (NM) has a positive, direct and significant effect on immigrant music attitude (MA) during quarantine period	0.003**	Significant
*Hypothesis 3b*: Native music (NM) has a positive, direct and significant effect on immigrants psychological wellbeing (PW) during Quarantine period	0.001***	Significant
*Hypothesis 4*: Music attitude (MA) has a positive, direct and significant effect on immigrant psychological wellbeing (PW) during Quarantine period	0.001***	Significant
*Hypothesis 5a*: Immigrants’ Music attitude (MA) positively mediates the relationship between music experience (MEX) and psychological wellbeing (PW) during Quarantine period	0.001***	Significant
*Hypothesis 5b*: Immigrants’ Music attitude (MA) positively mediates the relationship between native music (NM) and psychological wellbeing (PW) during Quarantine period	0.002**	Significant
*Hypothesis 6a*: Music type (MT) positively moderates the mediating relationship between music experience (MEX) and psychological wellbeing (PW) through music attitude during COVID quarantine period	0.241	Not Significant
*Hypothesis 6b*: Music type (MT) positively moderates the mediating relationship between native music (NM) and psychological wellbeing (PW) via music attitude during COVID quarantine period	0.187	Not Significant

Significance threshold values: ^†^*p* < 0.100, *p < 0.050, **p < 0.010, ***p < 0.001 (Gaskin et al., 2019).

Finally, the value of R^2^ for all independent constructs (MEX, SPI, and NM) on MA is 0.46. Furthermore, the author identified the value of R^2^ on PW by all independent, mediating, and moderating variables, such as MEX, SPI, NM, MT, and MA, as 0.46.

## 5. Discussion

Reaffirming the research objective of examining the precursors and consequences of music attitude among immigrants during the COVID quarantine period using the social cognitive theory, this study aims to explore how music experience, social media peer influence, native music, music type, and music attitude affect mood and emotion regulation and an individual’s wellbeing. This paper thoroughly analyzes these effects and highlights how a positive attitude toward music can promote psychological wellbeing during quarantine. The study’s results reveal some interesting findings.

First, during the COVID quarantine individuals’ motivation and attraction toward music listening, downloading, storing, sharing, etc. is dependent on their previous music experience, which also influenced their mental health and wellbeing. Hence, the authors may extrapolate that during the COVID quarantine period, international communities or immigrants tend to attain psychological wellbeing based on their positive attitude, which is influenced by their music experience. As in this study, the authors taken MEX as an essential cognitive factor that influences an individual’s music attitude and psychological wellbeing. Generally, in previous, studies, the relationship between individuals’ music experience, music attitude, and psychological wellbeing has been ignored (Musgrave, 2022), particularly during COVID-quarantine. Whereas a few other scholars, i.e., Du and Leung (2022) have conducted a qualitative study regarding the experience of music learning in a diverse community in Xinjiang, China. However, the authors of this study found limited studies about the effect of individuals’ music experiences on music attitudes, which may further influence his or her psychological wellbeing, particularly during the quarantine period. In contrast, a few researchers have investigated the emotional experiences, cognitive processes, and social aspects of music engagement among fans of violent rap, extreme metal music, and nonviolent Western classical music (Thompson et al., 2019; Olsen et al., 2022).

Second, this study posits that the individuals’ attitudes regarding music listening and music preferences were greatly influenced by their social media friend circles during quarantine period. In previous literature, the authors found limited content regarding the individuals’ social peer influence and music attitude well-being relationship. At the meantime, scholars highlighted that, people share their quarantine experiences on social media, demonstrating how they defeated the virus and maintained a healthy lifestyle during the difficult period of COVID quarantine (Vandenberg et al., 2021; Latikka et al., 2022).

Third, NM, which is taken as a cultural factor in this study, possessed substantial effect on MA and PW, respectively. It means that during the quarantine period, the immigrant’s music attitude, his musical patterns, and well-being is much more influenced by his native music. Because it is a human instinct, he usually prefers to listen to music in his native language because he feels and understands the music lyrics, music tune, and rhythm. Due to this reason and based on the research findings, the authors of this study claimed that native music or music in the native language could be considered as an influential cultural factor that may positively stimulate individuals’ music attitudes and psychological wellbeing; particularly during lockdown, isolation, and COVID quarantine situations. Whereas, Reyes-Martínez (2021) argued that cultural factors, i.e., musical concerts and community celebrations have not a substantial effect on psychological well-being of indigenous community. The authors of this study found limited studies examining the effect of cultural factors—native music in predicting an individual’s music attitude and psychological wellbeing. As Du and Leung (2022), while investigating the sustainability of music education in a specific area (where the “Miao” community used to live in China), claimed that Miao traditional music, gained greater respect because, of the lyrics in their native language and were an expression of their positive willingness to learn native music. Ivković (2013) revealed that instrumental music or songs in a language other than one’s own tends to evoke unfavorable attitudes and sentiments in a musical setting. Bodner and Bergman (2017), while analyzing the effectiveness of national music in reducing prejudice and enhancing theory of mind among Jews and Arabs in Israel, stated that songs or music in the native language, which may be the centerpiece of in-group favoritism, can be used to promote theory of mind even among opposing groups.

Fourth, in this study MA has acknowledged and have a positive, direct, and significant effect on PW, it means there are several essential cognitive, environmental, and cultural precursors that can positively influence an individual’s attitude toward music, and this positively stimulated music can further lead to individuals’ mental wellbeing and peacefulness. In previous literature, the association between music attitude and wellbeing has been taken as a source of self-enhancement Elvers et al. (2018). These scholars have considered a positive music listening attitude as empowering agent to stimulate both implicit and explicit self-esteem simultaneously.

Fifth, this study signifies that during COVID or quarantine time, there are certain different factors, i.e., MEX (cognitive/personal), SPI (environmental), and NM (cultural), which may collectively influence individuals’ mental peacefulness/psychological wellbeing via their attitude toward music. Generally, the author found scarce studies that examine the combined effect of cognitive (Rose et al., 2019; Manolika and Baltzis, 2022), environmental (Susino and Schubert, 2017; Elvers et al., 2018), and cultural factors Musgrave (2022) on individuals’ music attitudes, which may bring forth positive stimulation in individuals’ psychological wellbeing, particularly during the COVID quarantine period.

Finally, based on the study findings, it can be concluded that during the COVID quarantine period, different types of music do not have a moderating or changing effect on an individual’s music attitude, mental calmness, or wellbeing. However, the author observed that there have been only a few studies that explored the relationship between music type, attitude, and its impact on an individual’s psychological state. Some scholars have conducted social experiments using music types like country and classical as independent variables to influence willingness to pay for utilitarian products and social identity (Flynn et al., 2022). However, in the meantime, those studies ignored the effect of music type on individual psychological well-being, particularly during quarantine, lockdown, or isolation time periods when an individual is required to stay alone and separate from friends, family, parents, and other preference groups.

## 6. Implications

### 6.1. Theoretical implications

This study has the following theoretical implications for research. Focusing on the outcomes of the music attitude, particularly during the COVID-19 quarantine period among immigrants. This study identifies how a set of diverse influential precursors, i.e., cognitive—music experience (MEX), environmental—social media peer influence (SPI), and cultural— native music (NM), which collectively influence immigrants’ music attitude that bring forth significant effects on psychological well-being during their quarantine time period.

Although there is a sufficient amount of research on the relationship between music attitudes and behaviors, the majority of it has been conducted within a specific community/nation (Rose et al., 2019; Biasutti et al., 2021). Besides, in general, there is little research on immigrants’ music attitudes, particularly the relationship between music attitude and wellbeing during COVID quarantine. As very limited studies have dealt with the relationship between music and cognitive wellbeing during the COVID-19 pandemic to overcome mental problems such as anxiety, stress, and depression (Terasawa et al., 2021; Vajpeyee et al., 2022).

In addition, this study is based on SCT. As social cognitive theory suggests that individuals learn and adopt their attitudes, beliefs, and behaviors by observing others and the consequences of their actions (Wang et al., 2021). In regards to music, this theory suggests that individuals develop their music preferences and attitudes through observing, modeling, and reinforcement from their social environment. This can include influences from social media peers, who can spread and reinforce music preferences through online networks. Native music, or the music a person was exposed to during their formative years, can also play a strong role in shaping music preferences. Additionally, attitudes toward music can be influenced by observing others’ attitudes and emotions, and the relationship between music and psychological well-being is also shaped by these attitudes and emotions. Thus, social cognitive theory provides a framework for understanding the complex interplay between music experience, social media peer influence, native music, music attitude, and psychological well-being. Therefore, in this research, SCT can help the researchers to link the gap between individuals’ music attitude and not only psychological/cognitive factors; but also factors from other domains, particularly factors from the e-environment and cultural domains related to the music-psychological wellbeing relationship. Because, such a nexus can develop and support a sound musical ecosystem, that can help individuals to attain mental satisfaction. As the study results pointed out, there is a correlation between immigrants’ music experience and their psychological wellbeing via their music attitude.

Furthermore, the authors discovered enough studies in which the relationships between music attitude and motivation toward music learning in schools, colleges, and universities were examined in normal situations but ignored other relationships—the nexus between music attitude, essential precursors of music attitude-behavior, and psychological wellbeing during a COVID-quarantine situation, particularly from a multicultural perspective (Ho, 2022; Nichols and Springer, 2022). Therefore, the authors of this study have taken the immigrants traveling to China as their research sample and considered them as multicultural respondents. Because the respondents are from various countries and have different nationalities, their cultural backgrounds are also diverse.

Moreover, the study’s research model bears strong empirical support for music attitude and psychological wellbeing, which presents that music experience (cognitive factor), social media peer influence (an environmental factor), and native music (a cultural factor) have significant and positive effects on the music attitude and psychological wellbeing of immigrants during the quarantine period.

Besides, this study considers social media peer influence, i.e. (the environmental factor), and native music, i.e. (the cultural factor) as the newly essential and required antecedents of music attitude in the modern digital and globalized world. Prior research has paid less attention to environmental and cultural factors and their impact on musical attitudes or intentions. However, this study adds to the literature on the effective music attitude association with psychological well-being by taking cultural and environmental factors into account as key demanding factors in addition to individuals’ cognitive factors.

Finally, to the best of the authors’ knowledge, this study will be considered one of those studies that examine music attitude and its essential predictors that can positively affect mental and psychological wellbeing during COVID quarantine, particularly from a multicultural perspective.

### 6.2. Practical implications

This study reveals that the music attitude (MA) is positively and significantly influenced by cognitive factors—music experience (MEX), environmental factors—social media peer influence (SPI), and cultural factors —native music (NM). Besides, the authors identified that there is a positive and significant mediating effect of immigrants’ music attitude (MA) between music experience-psychological well-being relationships and native music and psychological well-being relationships. Whereas there is no significant moderated mediation effect of music type during the COVID quarantine time period. Therefore, based on the above findings, the authors put forward the following implications for music-related practitioners and the music industry:

First, music providers need to arrange one-click awareness online events about their productions and lyrics, and from time to time, the music providers need to arrange live streaming sessions to inform their audience regarding their new developments and what they will going to launch in the nearest future. Such events and online seminars will enhance individuals’ expertise and may update their music experience through frequent involvement in music-related updates and developments. Such events will also affect individuals’ preference groups (parents, family members, etc.), indirectly. Even the music providers can reap the benefits of social media advertisement time and again in a universal language to boost individuals’ music experiences.

Second, the music industry needs to strengthen peer involvement by producing a variety of music albums for different age groups, different interest groups and even providing the music lyrics with multiple language translation facilities. Such measures will increase music-exchanging patterns among immigrants, which will increase their involvement in musical activities. This will indirectly positively influence their musical attitudes and further their psychological well-being.

Thirdly, the music providers need to offer culturally diverse music to their target audience with the help of different electronic platforms, if they (music providers and producers) cannot handle the majority of the world’s music, at least they can provide the world’s popular music, e.g., Western music, Chinese music, Arabic music, Central Asian music, French music, etc., along with translation options if it comes to song domain.

Fourth, music practitioners must create specific, hassle-free, and user-friendly platforms where people can easily find music based on their knowledge, experience, and taste. Because, as of right now, most individuals know that they can find the music they want on platforms like YouTube, Facebook, YuKou, and others. However, these platforms are full of different things because they also offer social networking and other forms of entertainment. Therefore, it will be a great development if the music producers and providers create a few specific platforms for only music-related content, including different instrumental tunes, globally accessible song collections, diverse lyrics, etc.

Fifth, the music industries need to develop joint ventures with the popular social media platforms, where they can easily provide musical content according to the demographic information of the individuals who are associated with those social media platforms, because such alliances will increase the individuals’ music attitude, which further positively stimulates their psychological well-being, particularly during stress, loneliness, and COVID quarantine situations.

## 7. Limitations and future research

Although this study is a fresh addition to the literature on music attitude, and its essential factors to stimulate immigrants’ psychological wellbeing during their isolation – COVID quarantine period, however there are still abundant opportunities for future research and limitations. First, in this research, the majority of our respondents were males as compared to females. In future research, it is recommended to have equal representation of both females and males. Second, the generalizability of the outcomes may be challenging as the authors of this study collected data from those immigrants (immigrants) who travel to China. Future studies could examine the music-attitude-mental wellbeing association by taking the research respondents who traveled to other countries during the existing COVID pandemic. Additionally, data collection for this study took place during the COVID-19 pandemic when travel restrictions to China were in place, resulting in limited opportunities for individuals to travel smoothly and frequently. Consequently, only a small number of universities permitted their postdoctoral fellows, PhD candidates, and other students, who make up the majority of the study sample. Therefore, it is suggested that future studies explore less-educated study populations. Third, this research investigates the factors and consequences of positive music attitude during COVID-quarantine, but future studies may conduct research by examining different music types’ sentiments, i.e., classic, pop, rock, and hip-hop music, and their effects on individuals’ cognitive well-being.

Fourth, the study findings may be biased because, like any study on human attitudes and mental satisfaction, human prejudice/bias cannot be neglected. However, in this study, the researchers made every possible effort to ensure that their own biases did not influence the findings. Therefore, inviting co-authors for their contributions is one of the finest ways to reduce the researcher’s biases, each co-author contributes his or her own results and conclusions, which have been collaboratively discussed and incorporated to produce more accurate results. Additionally, conducting an empirical study using a mixed method (by combining quantitative and qualitative data from public agencies, statistical bureaus, data banks etc.) may reveal previously undiscovered insights about the subtleties of music attitude-behavior sentiments and their consequent effects on psycho-social wellbeing during an emergency situation, such as the COVID-19 pandemic.

Future research may also look at other aspects, such as age, the type of online music platform, personality attributes, parenting styles, and schooling factors, etc., which may have a significant impact on individuals’ music attitude and its consequences.

## 8. Conclusion

After the worldwide spread of COVID, psychological well-being based on individuals’ music attitude has received great attention and importance. Using social cognitive theory (SCT), this study aims to investigate the potential precursors of music attitude and assess their effects on the psychological well-being of immigrants, particularly during the COVID quarantine period. The findings of this study indicate that potential predictors such as cognitive-music experience (MEX), environmental-social media peer influence (SPI), and cultural factors-native music (NM) have a direct, significant, and positive effect on music attitude (MA), which further influences immigrants’ psychological well-being (PW) during their quarantine period. Additionally, the mediator (MA) mediates the relationships between MEX and PW and NM and PW, which are positive, significant, and regarded as partial mediation. However, the moderated mediation effects of music type (MT) on MEX-MA-PW and NM-MA-PW were found to be statistically insignificant. The findings of this study contribute to the music literature, particularly in the music attitude domain, by examining the essential precursors that stimulate immigrants’ music attitude, which further influence their psychological well-being during their quarantine period. This study provides substantial empirical evidence to support the findings and contributes to theory and practice concerning musical attitude, intentions, and associated psychological mental calmness during COVID quarantine. Therefore, the results of this study are likely to be a valuable addition to the music attitude-behavior literature, especially in the field of music psychological practices based on individuals’ music experience, social media peer influence, and their native music. Besides, this study will help practitioners to gain a better understanding of the significant relationships between immigrants’ psychological well-being and their attitudes and intentions toward music in health emergency situations, particularly during isolation time periods. The researchers hope that this study will provide new insights for scholars who are interested in understanding immigrants’ music attitudes and behaviors to achieve mental peace in health emergency situations.

## Data availability statement

The raw data supporting the conclusions of this article will be made available by the authors, without undue reservation. Requests to access the dataset should be directed to the corresponding author.

## Ethics statement

Ethical review and approval was not required for the study on human participants in accordance with the local legislation and institutional requirements. Written informed consent from the (patients/participants OR patients/participants legal guardian/next of kin) was not required to participate in this study in accordance with the national legislation and the institutional requirements.

## Author contributions

XW and WH collaboratively prepared this study. XW oversaw data collection and writing draft. WH conducted the data analysis, assisted XW to draft initial versions of the manuscript. Therefore, both authors collaborated to approved the final version of the manuscript.

## Conflict of interest

The authors declare that the research was conducted in the absence of any commercial or financial relationships that could be construed as a potential conflict of interest.

## Publisher’s note

All claims expressed in this article are solely those of the authors and do not necessarily represent those of their affiliated organizations, or those of the publisher, the editors and the reviewers. Any product that may be evaluated in this article, or claim that may be made by its manufacturer, is not guaranteed or endorsed by the publisher.
